# Fluoride-Mediated Synthesis of Co(OH)F and Electronic Structure Optimization for Enhanced Water Oxidation Performance

**DOI:** 10.3390/molecules30173529

**Published:** 2025-08-29

**Authors:** Qianqian Dong, Yuhao Li, Jihao Liu, Yaru Wen, Junjie Wang, Haining Mo, Qianqian Jin, Shaohui Zhang, Xiong He

**Affiliations:** Guangxi Key Laboratory of Multidimensional Information Fusion for Intelligent Vehicles, Guangxi Colleges and Universities Key Laboratory of Microwave Communication and Micro–Nano Photoelectric Technology, School of Electronic Engineering, Guangxi University of Science and Technology, Liuzhou 545000, China; isdongqq@163.com (Q.D.); q2476166290@126.com (Y.L.); 15356856860@163.com (J.L.); 13906438619@163.com (Y.W.); m18437649108@163.com (J.W.); qqjin10s@139.com (Q.J.); zhangshaohui@gxust.edu.cn (S.Z.)

**Keywords:** fluorine anion, basic cobalt salt, electronic structure modulation, oxygen evolution reaction

## Abstract

This study deciphers the anionic modulation mechanism of halide ions (F^−^/Cl^−^) in cobalt-based hydroxides for oxygen evolution reaction (OER). Phase-pure Co(OH)_2_, Co(OH)F, and Co_2_(OH)_3_Cl were fabricated via substrate-independent hydrothermal synthesis to eliminate conductive support interference. Electrocatalytic evaluation on glassy carbon electrodes demonstrates fluoride’s superior regulatory capability over chloride. X-ray photoelectron spectroscopy (XPS) analyses revealed that F^−^ incorporation induces charge redistribution through Co → F electron transfer, optimizing the electronic configuration via ligand effects. F^−^ incorporation simultaneously guided the anisotropic growth of 1D nanorods and reduced surface energy, thereby enhancing the wettability of Co(OH)F. The engineered Co(OH)F catalyst delivers exceptional OER performance: 318 mV overpotential at 10 mA/cm^2^ in 1 M KOH with 94% current retention over 20 h operation. This study provides a synthetic strategy for preparing pure-phase Co(OH)F and compares halide ions’ effects on enhancing OER activity through electronic structure modulation and morphological control of basic cobalt salts.

## 1. Introduction

OER is an anodic half-reaction essential for energy-conversion technologies, involving kinetically sluggish four-electron transfer and O–O bond formation [1,2,3]. The development of efficient catalysts is critically important for overcoming kinetic barriers [4,5,6]. Fe-, Co-, and Ni-based hydroxides are representative non-precious-metal-based OER electrocatalysts, exhibiting facile preparation and high activity [7,8,9,10]. Among them, the exceptional catalytic activity of Co is primarily due to its 3d^7^ electron configuration, facilitating multivalent states (Co^2+^, Co^3+^, Co^4+^) [11,12,13,14]. For this, Co readily combines with other elements or substrates to form composites that modulate electron transfer, providing a diversity of active sites [15,16]. Research on Co-based catalysts requires further in-depth exploration of optimization strategies and mechanisms.

The basic cobalt salts Co_x_(OH)_y_A_z_ serve as a high-efficiency anode catalyst for alkaline water electrolysis, in which the anion plays a crucial role in structural regulation [17,18]. Xu et al. synthesized Co(OH)(CO_3_)_0.5_, Co_2_(OH)_3_Cl, and Co(OH)F alkaline cobalt salt catalysts on nickel foam (NF) for OER and investigated the anion effect [19]. Chen et al. prepared the FCoOOH/NF catalyst, which features a weak metal–fluorine (F) bond with strong ionicity. This bond facilitated the dynamic migration of fluoride anions, significantly improving the OER activity and reaction kinetics of the cobalt-based catalyst [20]. In addition, Co(OH)F is synthesized alongside other Ni-, Fe-, and Co-based oxides/sulfides to form heterostructure catalysts, with F^−^ regulating electron transfer at the heterointerface [18,21,22]. Zha et al. synthesized chlorine (Cl)-doped α-Co(OH)_2_ exhibiting excellent OER activity, where chloride-induced etching triggered defect formation in the catalyst [23]. Kou et al. grew Cl-doped Co(OH)_2_ in situ on carbon fiber cloth (CF), exhibiting enhanced OER activity and durability due to defect structure with an enlarged electrochemically active surface area formed during electrochemical oxidation [24]. Meng et al. considered that the formation of Co_2_(OH)_3_Cl microstructures was a complex process, and proposed a new method for synthesizing Co_2_(OH)_3_Cl in the eggshell reactor system for lithium-ion batteries [25]. F^−^ and Cl^−^ are demonstrated as electronic modulators for Co-based OER catalysts across these studies. However, most of the studies are conducted by compounding other metal oxides or hydroxides on NF, and the synthesis method is relatively complicated [26]. At present, systematic research on the effects of halide ions (F^−^ and Cl^−^) on the Co_x_(OH)_y_A_z_ for OER remains limited.

To examine the influence of halide ions on Co_x_(OH)_y_A_z_, pure Co(OH)F, Co_2_(OH)_3_Cl, and Co(OH)_2_ were synthesized by a simple one-step hydrothermal method avoiding interference from NF substrates. The OER activities of F^−^/Cl^−^-modified Co(OH)_2_ were comparatively evaluated on glassy carbon electrodes. As the most electronegative anion, F^−^ exerts a greater regulating effect on Co(OH)_2_ than Cl^−^. F^−^ incorporation regulates electron transfer from Co to F, optimizing the electronic structure of Co(OH)F. Additionally, F^−^ introduction induces the formation of interlaced 1D nanorods in Co(OH)F and reduces surface free energy, thereby enhancing material wettability. Co(OH)F requires an overpotential of only 318 mV to achieve 10 mA/cm^2^ in 1 M KOH, maintaining 94% current retention over 20 h at this current density. Our study has important implications for the design of halide ion-mediated OER catalysts for alkaline cobalt salts.

## 2. Result and Discussion

### 2.1. Morphology and Structure Characterization

**Figure 1:**
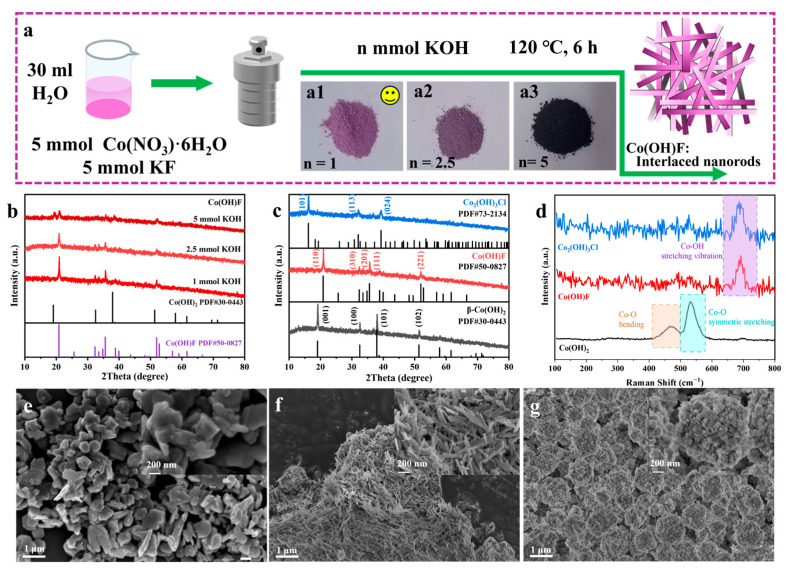
(**a**) Schematic illustration of the preparation processes of Co(OH)F: photo images of (**a1**) 1 mmol, (**a2**) 2.5 mmol, and (**a3**) 5 mmol KOH that were used. (**b**) XRD patterns of Co(OH)F at different KOH dosage. (**c**) XRD patterns and (**d**) Raman spectra of Co(OH)_2_, Co(OH)F, and Co_2_(OH)_3_Cl samples. SEM images of (**e**) Co(OH)_2_, (**f**) Co(OH)F, and (**g**) Co_2_(OH)_3_Cl.

The simple one-step hydrothermal synthesis route of Co(OH)F is schematized in Figure 1a, where a1, a2, and a3 are optical photographs of the synthesized products, exhibiting pinkish purple, dark purple, and black coloration corresponding to 1, 2.5, and 5 mmol KOH additions, respectively. The X-ray diffraction (XRD) results in Figure 1b confirm the synthesis of pure Co(OH)F using 1 and 2.5 mmol KOH. Distinct characteristic diffraction peaks at 2θ = 20.8°, 32.3°, 33.5°, 35.6°, and 51.9° correspond to the (110), (310), (201), (111), and (221) crystallographic planes of Co(OH)F, respectively (PDF#50-0827) [27,28,29]. In contrast to the pure Co(OH)F phases above, the use of 5 mmol KOH resulted in a distinct diffraction peak at 2θ = 19.1°. This peak corresponds to the (001) plane of β-Co(OH)_2_ (PDF#30-0443) [30], confirming the formation of Co(OH)F contaminated with β-Co (OH)_2_ impurity. Pure β-Co(OH)_2_ and Co_2_(OH)_3_Cl were similarly synthesized using 1, 2.5, and 5 mmol KOH. Figure S1 presents their optical images and XRD patterns. β-Co(OH)_2_ exhibited black coloration, accounting for the progressive darkening of Co(OH)F products (from pinkish purple to black) with elevated KOH concentrations. This originates from β-Co(OH)_2_ impurity formation under excess KOH conditions. To maintain experimental consistency, the samples synthesized with 1 mmol KOH were selected for further study (Figure 1c). The diffraction peaks appearing at 2θ = 19.1°, 32.5°, 37.9°, and 51.4° represent the (001), (100), (101), and (102) crystallographic planes of β-Ch(OH)_2_, respectively (PDF#30-0443) [13,28]. Peaks at 2θ = 16.2°, 32.0°, and 39.2° are assigned to the (101), (113), and (024) planes of Co_2_(OH)_3_Cl, respectively (PDF#73-2134) [25]. Figure 1d shows the Raman spectrum of samples. The peaks at approximately 470, 520, and 680 cm^−1^ are attributed to vibrational modes of the cobalt species: Co-O bending, Co-O symmetric stretching and Co-OH stretching vibration, respectively [31,32]. The morphology of the samples was analyzed using scanning electron microscopy (SEM). Co(OH)_2_ exhibits irregular block-like agglomerates (Figure 1e). Co(OH)F displays interlaced one-dimensional (1D) nanorods (Figure 1f). Co_2_(OH)_3_Cl shows hydrangea-like microspheres composed of cubic and spherical agglomerates (Figure 1g). This series of morphological evolutions clearly demonstrates the key roles of F^−^ and Cl^−^ as morphology-directing agents. F^−^ is an effective regulator for inducing 1D anisotropic growth [17,33], while Cl^−^ tends to promote the 3D structure formed by cubic and spherical assembly [23].

**Figure 2:**
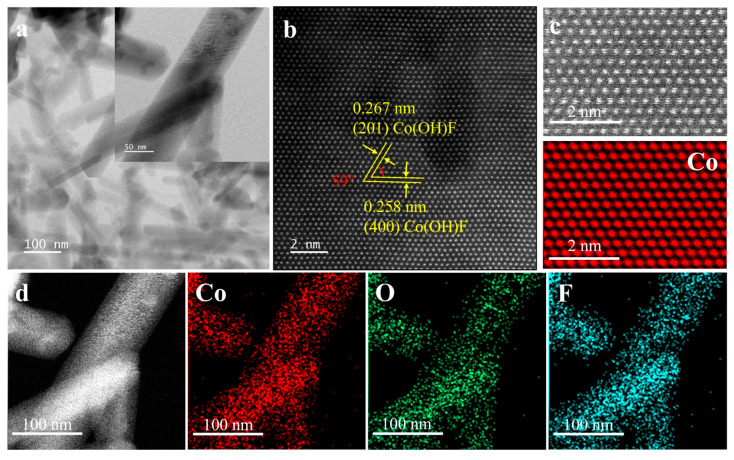
(**a**) TEM images and (**b**) HRTEM image of Co(OH)F. (**c**,**d**) EDS elemental mapping images of Co(OH)F.

Transmission electron microscopy (TEM) is used to characterize the microscopic morphology and crystal structure of materials. Figure 2a shows that Co(OH)F is composed of interspersed one-dimensional nanorods, which is consistent with the previous SEM results. The lattice stripes with spacing of 0.267 and 0.258 nm are indexed to the (201) and (400) planes of Co(OH)F, respectively. The angle between these two crystal planes is 59°, which is consistent with the crystallographic parameters of the orthorhombic crystal structure (Figure 2b) [17,34]. Atomic-resolution energy-dispersive X-ray spectroscopy (EDS) clearly reveals the distribution of Co atoms (Figure 2c). The EDS elemental mapping in Figure 2d shows the homogeneous dispersion of Co, O, and F across the nanorods. Figure S2a–c demonstrate that Co_2_(OH)_3_Cl exhibits the structure of square and circular nanosheet agglomerates, consistent with the SEM results. The lattice stripes with spacing of 0.290 and 0.214 nm correspond to the (021) and (122) crystallographic planes of Co_2_(OH)_3_Cl, respectively (Figure S2d). The EDS mapping reveals uniform distribution of Co, O, and Cl elements (Figure S2e), indicating the successful doping of the Cl element. The atomic ratios of Co, O, and F/Cl in Co(OH)F and Co_2_(OH)_3_Cl were determined by EDS spectra analysis (Figure S3). The results reveal that the Co:O:F ratio in Co(OH)F is approximately 1.0:0.73:0.97, which closely matches the theoretical stoichiometry of 1:1:1. Similarly, the Co:O:Cl ratio in the selected region of Co_2_(OH)_3_Cl is approximately 3.1:2.0:1.0. The ratio of introduced Cl to (Co+O) is 1:5.1, which agrees with the theoretical value of 1:5.

**Figure 3:**
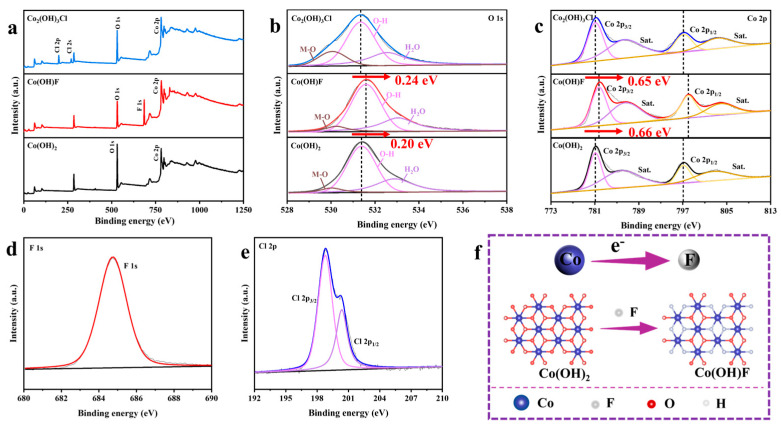
(**a**) XPS survey spectra. (**b**) O 1s and (**c**) Co 2p spectra of Co(OH)_2_, Co(OH)F, and Co_2_(OH)_3_Cl. (**d**) F 1s spectrum of Co(OH)F. (**e**) Cl 2p spectrum of Co_2_(OH)_3_Cl. (**f**) Schematic diagram of electron transfer.

X-ray photoelectron spectroscopy (XPS) characterization reveals the surface elemental composition and chemical states of the samples. XPS spectra were calibrated using the C 1s peak at 284.8 eV [35,36]. Figure 3a presents XPS survey spectra of Co(OH)_2_, Co(OH)F, and Co_2_(OH)_3_Cl. Distinct F 1s and Cl 2s/2p peaks observed in Co(OH)F and Co_2_(OH)_3_Cl spectra provide evidence for successful halogen incorporation into the cobalt hydroxide lattice. Figure 3b shows the O 1s spectra deconvoluted into three peaks of M-O, O-H, and H_2_O. The O-H peaks for Co(OH)_2_, Co(OH)F, and Co_2_(OH)_3_Cl are located at 531.39, 531.59, and 531.35 eV, respectively [18,27]. Compared with Co(OH)_2_ and Co_2_(OH)_3_Cl, the O-H peak in Co(OH)F shifts to higher binding energy by 0.20 and 0.24 eV, respectively. The observed O-H binding energy shifts imply reduced electron density around O in Co(OH)F. In the Co 2p spectra, the peaks at 781.09 and 797.09 eV are attributed to Co 2p_3/2_ and 2p_1/2_ of Co(OH)_2_, respectively [31]. The corresponding peaks of Co_2_(OH)_3_Cl are located at 781.10 eV (Co 2p_3/2_) and 797.12 eV (Co 2p_1/2_). For Co(OH)F, the Co 2p_3/2_ and 2p_1/2_ binding energies are at 781.75 and 798.09 eV, respectively [19]. The Co2p_1/2_ peak in Co(OH)F exhibits the positive binding energy shifts of 0.66 and 0.65 eV compared to Co(OH)_2_ and Co_2_(OH)_3_Cl, indicating electron loss at Co sites induced by F incorporation. Figure 3d shows that the F 1s peak in Co(OH)F appears at 684.72 eV [29]. For Co_2_(OH)_3_Cl, the two peaks at 198.89 and 200.46 eV correspond to Cl 2p_3/2_ and 2p_1/2_, respectively (Figure 3e) [37]. According to the XPS analysis, the addition of F regulates electron transfer from Co to F and modulates the electronic structure of Co(OH)F, as shown in Figure 3f.

### 2.2. Electrochemical Performance

**Figure 4:**
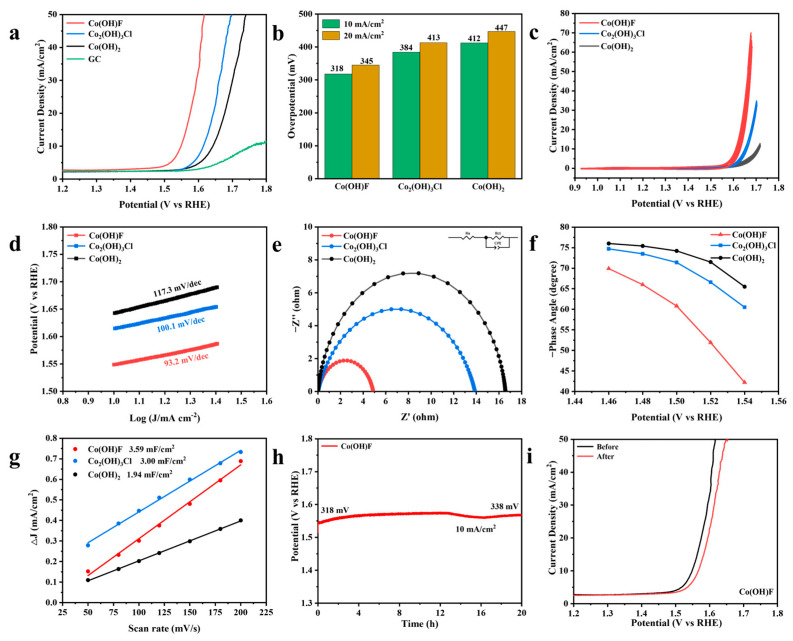
OER performance of Co(OH)F, Co_2_(OH)_3_Cl, and Co(OH)_2_ in 1 M KOH solution. (**a**) LSV curves with *iR* correction. (**b**) Comparison of the overpotentials required to achieve 10 and 20 mA/cm^2^. (**c**) CV activation curves (**d**) Tafel plots. (**e**) Nyquist plots. (**f**) Potential dependence of the phase angles. (**g**) Double-layer capacitance. (**h**) CP curve of Co(OH)F at 10 mA/cm^2^ for 20 h. (**i**) Comparison of the LSV curves before and after CP test.

The OER catalytic performance of Co(OH)F, Co_2_(OH)_3_Cl, and Co(OH)_2_ was evaluated using a three-electrode system in 1 M KOH. In Figure 4a,b, Co(OH)F exhibits the best catalytic performance, requiring only 318 and 345 mV overpotentials to reach current densities of 10 and 20 mA/cm^2^, respectively. Co_2_(OH)_3_Cl and Co(OH)_2_ require 384/413 and 412/447 mV overpotentials to reach 10/20 mA/cm^2^, respectively. The OER performance of Co_x_(OH)_y_A_z_ synthesized by F^−^ regulation is better than that of Cl^−^. Figure 4c shows the cyclic voltammetry (CV) activation curves of the samples before testing the linear sweep voltammetry (LSV), and the good overlap indicates adequate activation. The smaller value of the Tafel slope indicates the faster kinetic of the catalyst [38]. Compared to Co_2_(OH)_3_Cl (100.1 mV/dec) and Co(OH)_2_ (117.3 mV/dec), Co(OH)F has the smallest Tafel slope of 93.2 mV/dec. This demonstrates that F^−^ regulation of Co_x_(OH)_y_A_z_ significantly accelerates the OER kinetics (Figure 4d). In the Nyquist plot (Figure 4e), Co(OH)F exhibits the smallest semicircle radius, indicating the lowest impedance in the reaction. Figure 4f shows the variation of the phase angle with potential over the range of 1.46 to 1.54 V, as derived from the Bode diagram of the electrocatalyst (Figure S4). Co(OH)F exhibits the lowest phase angle, indicating more electron transfer during the OER compared to other catalysts, thereby increasing the reaction rate [39]. As a crucial metric for the specific surface area of catalyst, the electrochemically active surface area (ECSA) exhibits a positive correlation with the double-layer capacitance (C_dl_) [40]. Figure 4g shows the C_dl_ derived from CV curves at various scan rates (Figure S5). The C_dl_ values for Co(OH)F, Co_2_(OH)_3_Cl, and Co(OH)_2_ are 3.59, 3.00, and 1.94 mF/cm^2^, respectively. The largest C_dl_ for Co(OH)F implies that it may provide more active surface area. ECSA values were computed as C_dl_ divided by the specific capacitance value of 40 µF/cm^2^ in 1 M KOH [21,41,42]. The ECSA-normalized LSV curves in Figure S6 demonstrate that Co(OH)F exhibits the highest inherent OER activity among the catalysts. Figure S7 compares the OER performance of electrodes prepared with an equal catalyst loading on nickel foam (1 cm × 0.5 cm) under identical conditions. Consistent with the results obtained using the GCE, the Co(OH)F/NF electrode exhibited the best OER performance, followed by Co_2_(OH)_3_Cl/NF. Figure 4h shows the chronopotentiometry (CP) curves for 20 h of operation at 10 mA/cm^2^. The LSV curves before and after the stability test exhibit minimal degradation in Figure 4i. XRD and SEM analyses were performed on the after-reaction (AR) materials. The XRD pattern of Co(OH)F-AR remains unchanged (Figure S8), indicating that the bulk phase of Co(OH)F is maintained after the OER long-term stability test, which is consistent with previous reports [19]. SEM images indicate that Co(OH)F-AR maintains its interlaced 1D nanorods morphology without severe corrosion (Figure S9). Co(OH)F’s electrocatalytic activity toward OER in this study was superior to that of related Co-based catalysts reported recently (Table S1).

**Figure 5:**
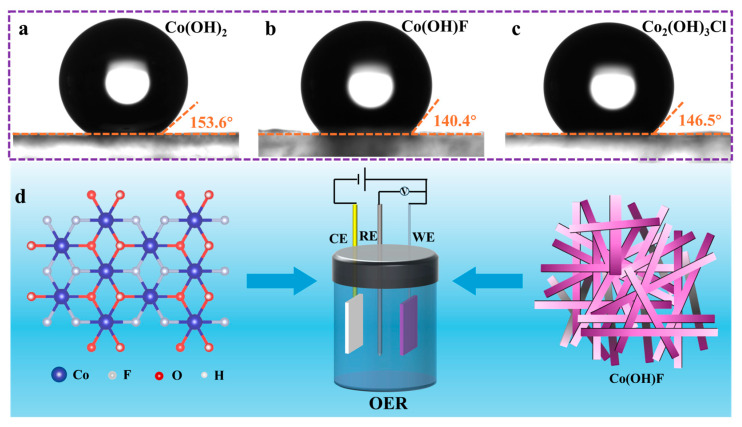
Static water contact angles of (**a**) Co(OH)_2_, (**b**) Co(OH)F, and (**c**) Co_2_(OH)_3_Cl. (**d**) Schematic diagram: optimizing electronic structure and morphology of Co(OH)F for OER performance.

An equal amount of the prepared slurry (500 μL) was uniformly coated on the conductive surface of FTO substrates (1 cm × 2 cm). The static water contact angles for Co(OH)_2_, Co(OH)F, and Co_2_(OH)_3_Cl are 153.6°, 140.4°, and 146.5°, as shown in Figure 5a–c. The incorporation of F^−^ and Cl^−^ enhanced the wettability compared to pure Co(OH)_2_, with Co(OH)F exhibiting optimal hydrophilicity. The wettability of a solid substrate is primarily governed by its surface energy and topography [43,44]. According to the SEM results in Figure 1f, Co(OH)F displays interlaced 1D nanorods which facilitate charge carrier transport [17]. F is the most electronegative element, and F^−^ exhibits strong polarizing power due to high charge density [20,45]. F^−^ incorporation modifies the electronic structure and surface energy of Co(OH)F, thereby enhancing its OER performance (Figure 5d).

## 3. Experimental Section

### 3.1. Materials and Chemicals

Cobaltous nitrate hexahydrate (Co(NO_3_)·6H_2_O) and potassium fluoride (KF) were purchased from Beijing InnoChem Science & Technology Co., Ltd. (Beijing, China). Potassium chloride (KCl) and potassium hydroxide (KOH) were purchased from Shanghai Aladdin Biochemical Technology Co., Ltd. (Shanghai, China). Nafion (5 wt%) was purchased from Tianjin Avexin Chemical Technology Co., Ltd. (Tianjin, China). Nickel foam (NF) with a thickness of 1.0 mm and a purity of 99.9% was purchased from Taiyuan Lizhiyuan Technology Co., Ltd. (Taiyuan, China). All chemicals met analytical-grade specifications and were utilized without additional processing.

### 3.2. Preparation of Electrode Materials

To begin, 5 mmol Co(NO_3_)_2_·6H_2_O, 5mmol KF, and different amounts of KOH (1, 2.5, and 5 mmol) were dissolved in 30 mL deionized water, then thoroughly sonicated until uniform dispersion. Subsequently, a 20 mL aliquot of this solution was added to a 20 mL Teflon-lined stainless-steel autoclave and hydrothermally treated at 120 °C for 6 h. Following cooling to ambient temperature, products were collected by 6000 rpm centrifugation, then washed sequentially three times with pure ethanol and deionized water. Finally, the collected samples were vacuum-dried (60 °C, 10 h) to eliminate volatile residues. Co_2_(OH)_3_Cl was synthesized using the solution containing 5 mmol KCl rather than KF. Co(OH)_2_ can be prepared by mixing solutions of Co(NO_3_)_2_·6H_2_O and KOH. Other conditions remain unchanged.

After polishing with 0.05 µm Al_2_O_3_, the glassy carbon electrode (GCE, 3 mm diameter) was ultrasonically rinsed sequentially in 1 M HCl, ethanol, and deionized water. A total of 5 mg Co(OH)F (prepared by adding 1 mmol KOH) was blended with 50 μL Nafion and 450 μL alcohol under sonication. Then 5 μL of the homogeneous slurry was coated onto the cleaned GCE using a pipette. After naturally air-drying, the Co(OH)F electrode was formed. The Co(OH)_2_ and Co_2_(OH)_3_Cl electrodes were prepared in the same way.

### 3.3. Characterization

XRD analysis was performed on a Rigaku SmartLab SE diffractometer (Tokyo, Japan) configured for operation at 40 kV and 40 mA. Monochromatic Cu Kα radiation (λ = 1.54178 Å) was utilized for pattern acquisition. Raman spectra (excitation wavelength of 532 nm) were recorded employing the Zhuolihanguang/RST2-301-SMS spectrometer (Beijing Zhuo Li Han Kuang Instrument Co., Ltd., Beijing, China). SEM images were captured with a ZEISS Sigma 360 microscope operating at 3 kV. TEM images and associated EDS mapping were conducted using the JEOL/JEM-ARM200F instrument (Tokyo, Japan). The microscope was operated at an accelerating voltage of 200 kV. XPS studies were carried out on a Thermo Scientific K-AIpha (Waltham, MA, USA). Static water contact angle measurements were defined by a Lauda Scientific LSA100 (Lauda-Königshofen, Germany). The test started at the moment when a 2 uL droplet was in contact with the surface of the material and remained stable.

### 3.4. Electrochemical Measurements

Electrochemical measurements utilized a standard three-electrode cell containing 1 M KOH electrolyte, performed at ambient temperature with a CH760E workstation (CH Instruments, Inc., Austin, TX, USA). The prepared Co(OH)F/Co_2_(OH)_3_Cl/Co(OH)_2_ electrodes were used as the working electrodes (WE). Hg/HgO and platinum served as the reference electrode (RE) and counter electrode (CE), respectively. Electrochemical activation of the electrocatalyst was performed by CV before conducting the main electrochemical tests with a scan rate of 50 mV/s over 20 cycles, covering the potential range of 0 to 0.8 V vs. Hg/HgO. Using a 5 mV/s scan rate and a potential window of 0 to 0.9 V vs. Hg/HgO, LSV curves of the electrocatalysts were recorded. Electrochemical impedance spectroscopy (EIS) was conducted in the frequency ranging from 10^5^ to 10^−1^ Hz at the potential of open-circuit voltage (OCV) vs. Hg/HgO. CP was tested at 10 mA/cm^2^ for 20 h. Throughout the electrochemical testing, measured potentials were converted to the reversible hydrogen electrode (RHE) scale via

$$E_{\mathrm{RHE}}={\;E}_{\mathrm{Hg}/\mathrm{HgO}\;}+0.0592\;\text{×}\;\mathrm{pH}+0.098$$

Overpotential values (η) were derived employing the expression below:

$$\eta \;=\;E_{\mathrm{RHE}}\;-\;1.23\;V$$

## 4. Conclusions

This study focused on the halide-mediated synthesis of pure Co_x_(OH)_y_A_z_ without conductive supports and investigated the mechanism by which halide ions enhance the OER activity of Co(OH)_2_. Pure Co(OH)_2_, Co(OH)F, and Co_2_(OH)_3_Cl were successfully prepared via the one-step hydrothermal method. F^−^ incorporation induces charge redistribution through Co → F electron transfer, optimizing the electronic configuration. This electronic reshaping simultaneously drives anisotropic growth to form interlaced 1D nanorods and reduces surface energy, thereby enhancing the wettability of Co(OH)F. The resulting electronic–structural synergistic effect imparts excellent OER performance to Co(OH)F: a minimum overpotential of 318 mV at 10 mA/cm^2^ and robust durability (activity decay < 6% after 20 h of operation). This work provides new insights into the halide-mediated synthesis of Co_x_(OH)_y_A_z_ electrocatalysts.

## Data Availability

The original contributions presented in this study are included in the article/Supplementary Material. Further inquiries can be directed to the corresponding author(s).

## Supplementary Materials

The following supporting information can be downloaded at https://www.mdpi.com/article/10.3390/molecules30173529/s1, Figure S1: photo images of samples of (a) Co(OH)_2_ and (b) Co_2_(OH)_3_Cl, and XRD patterns of samples of (c) Co(OH)_2_ and (d) Co_2_(OH)_3_Cl; Figure S2: (a–c) TEM images of Co_2_(OH)_3_Cl, (d) HRTEM image of Co_2_(OH)_3_Cl, and (e) EDS elemental mapping images of Co_2_(OH)_3_Cl; Figure S3: EDS spectra and corresponding atomic ratios of (a) Co(OH)F and (b) Co2(OH)3Cl; Figure S4: Bode plots of (a) Co(OH)F, (b) Co_2_(OH)_3_Cl, and (c) Co(OH)_2_ at potential range from 1.46 to 1.54 V vs. RHE; Figure S5: CV at different scan rates (from 50 to 200 mV/s) for (a) Co(OH)_2_, (b) Co(OH)F, and (c) Co_2_(OH)_3_Cl; Figure S6: ECSA-normalized LSV curves with iR correction; Figure S7. LSV curves of Co(OH)F, Co2(OH)3Cl and Co(OH)2-loaded nickel foam with iR correction; Figure S8. XRD pattern of Co(OH)F after OER; Figure S9. (a,b) SEM images of Co(OH)F after OER. Table S1: comparison of the OER performance of Co(OH)F catalyst in 1 M KOH.
