# Supplementary material for: Fluoride-Mediated Synthesis of Co(OH)F and Electronic Structure Optimization for Enhanced Water Oxidation Performance
Source: Molecules. 2025 Aug 29;30(17):3529. doi: 10.3390/molecules30173529 (PMC12430501; doi:10.3390/molecules30173529)
Supplement: Supplementary file 1 [file molecules-30-03529-s001.zip › molecules-3830706-supplementary.pdf]

# Fluoride-Mediated Synthesis of Co(OH)F and Electronic Structure Optimization for Enhanced Water Oxidation Performance

*Qianqian Dong, Yuhao Li, Jihao Liu, Yaru Wen, Junjie Wang, Haining Mo, Qianqian Jin, Shaohui Zhang, Xiong He*

*Guangxi Key Laboratory of Multidimensional Information Fusion for Intelligent Vehicles, Guangxi Colleges and Universities Key Laboratory of Microwave Communication and Micro－Nano Photoelectric Technology, School of Electronic Engineering, Guangxi University of Science and Technology, Liuzhou 545000, China.*

*Corresponding author: Tel.: +86-0451-86282153.

E-mail address: hnmo@163.com (H. Mo), hexiong@gxust.edu.cn (X. He).

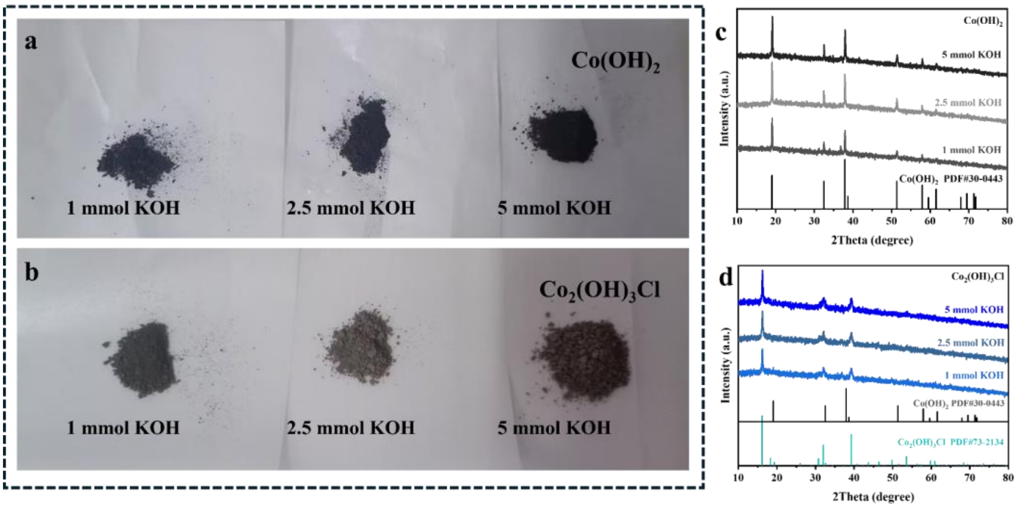


**Figure S1.** Photo images of samples. (a) $Co(OH)_2$ and (b) $Co_2(OH)_3Cl$. XRD patterns of samples. (c) $Co(OH)_2$ and (d) $Co_2(OH)_3Cl$.

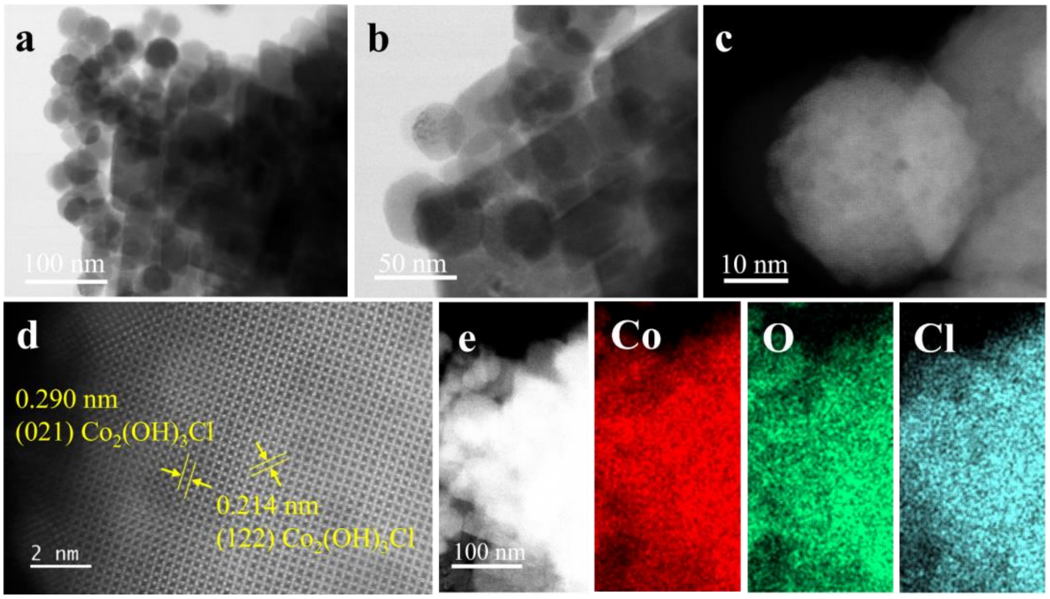


**Figure S2.** (a-c) TEM images of $Co_2(OH)_3Cl$, (d) HRTEM image of $Co_2(OH)_3Cl$ and (e) EDS elemental mapping images of $Co_2(OH)_3Cl$.

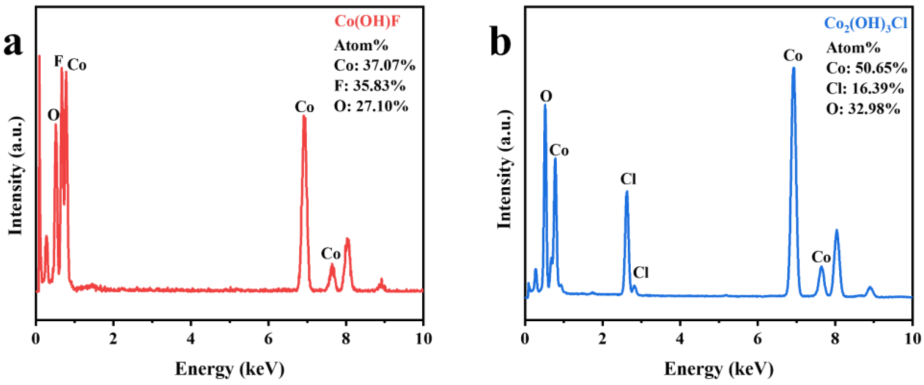


**Figure S3.** EDS spectra and corresponding atomic ratios of (a) Co(OH)F and (b) $Co_2(OH)_3Cl$

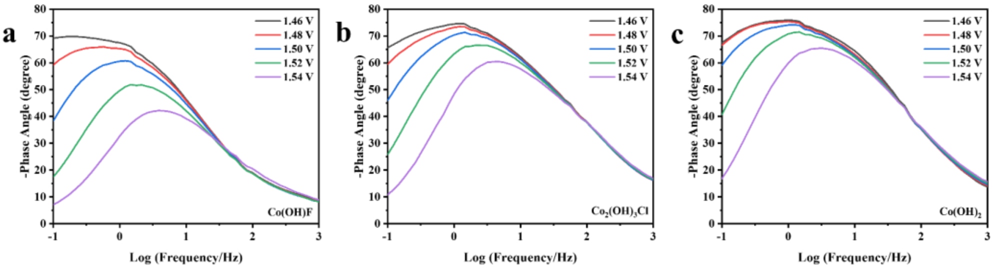


**Figure S4.** Bode plots of (a) Co(OH)F, (b) $Co_2(OH)_3Cl$ and (c) $Co(OH)_2$ at potential range from 1.46 to 1.54 V vs RHE.

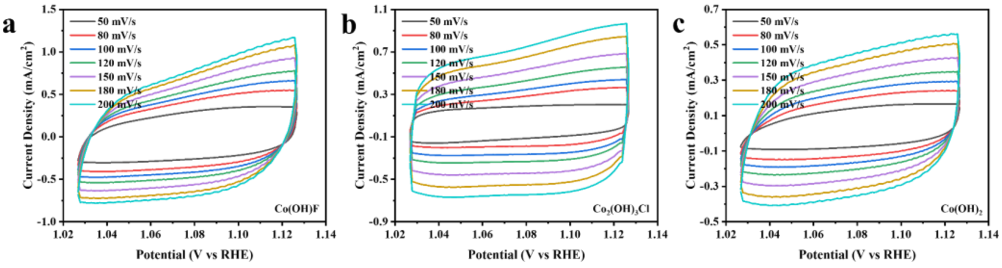


**Figure S5.** CV at different scan rates (from 50 to 200 mV/s). (a) $Co(OH)_2$, (b) Co(OH)F and (c) $Co_2(OH)_3Cl$.

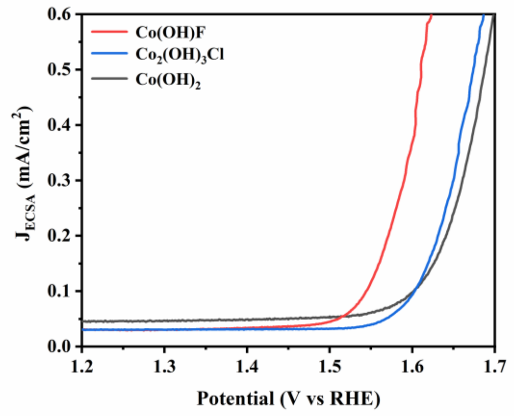


**Figure S6.** ECSA-normalized LSV curves with *iR* correction.

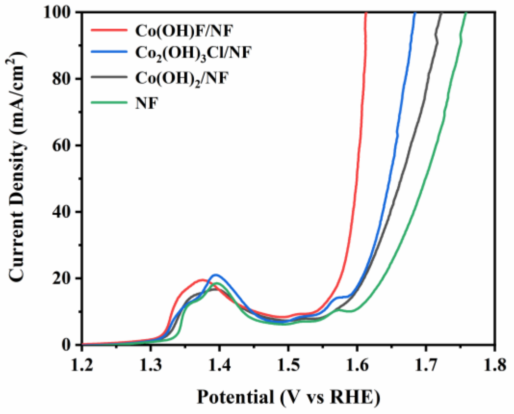


**Figure S7.** LSV curves of Co(OH)F, $Co_2(OH)_3Cl$ and $Co(OH)_2$-loaded nickel foam with *iR* correction.

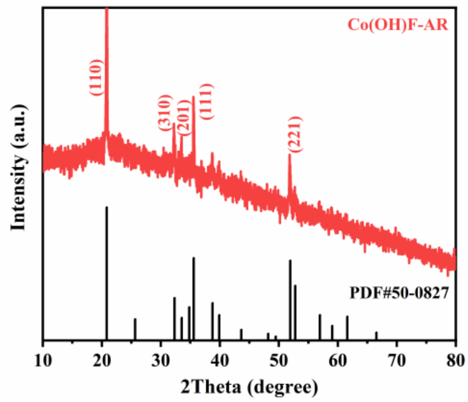


**Figure S8.** XRD pattern of Co(OH)F after OER.

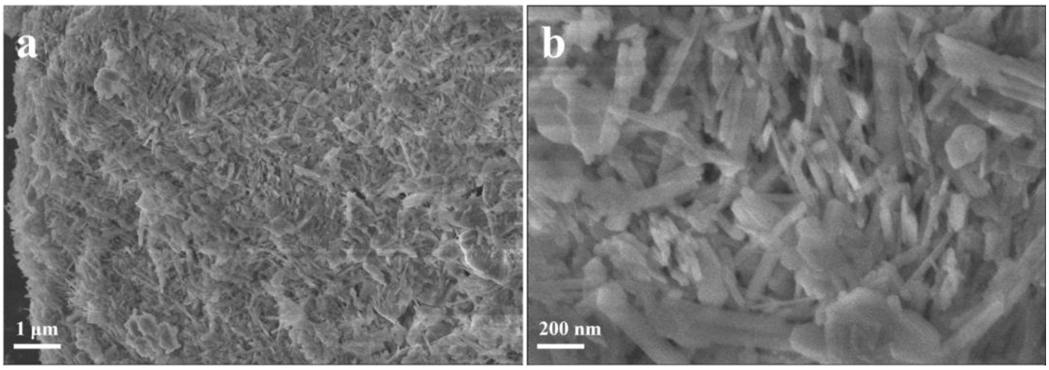


**Figure S9.** (a-b) SEM images of Co(OH)F after OER.

**Table S1.** Comparison of the OER performance of Co(OH)F catalyst in 1 M KOH.

| Electrocatalysts | $J$ $mA/cm^2$ | $\eta$ (mV) | Tafel slope (mV/dec) | Stability | Reference |
|---|---|---|---|---|---|
| **Co(OH)F** | **10**<br>**20** | **318**<br>**345** | **93.2** | **20 h at 10 $mA/cm^2$** | **This work** |
| $CoF_2$<br>$NiF_2$ | 10<br>10 | 355<br>370 | 47.3<br>54.2 | ~<br>~ | Adv. Sci. 2023, 2306758 |
| Co(OH)F<br>$Co_9S_8$/Co(OH)F | 10<br>10 | 358<br>329 | 102<br>87 | ~<br>~ | New J. Chem. 2022, 46, 23060-23065 |
| $Co_2P/Co_2N$@CNF-DNA(C) | 10 | 360 | 107.42 | 14 h at 10 $mA/cm^2$ | Electrochim. Acta 2021, 367, 137562 |
| $Fe_3O_4$–$CoP_x$/TiN | 10 | 331 | 122 | 11 h at 10 $mA/cm^2$ | ACS Appl. Nano Mater. 2019, 2, 1, 40–47 |
| EO/Cl-doped $Co(OH)_2$ | 10 | 330 | 98 | 12 h at 10 $mA/cm^2$ | ACS Appl. Mater. Interfaces 2018, 10, 796−805 |
| β-$Co(OH)_2$/ Co(OH)F | 10 | 329 | 60.5 | 10 h i-t test | Electrochim. Acta 2018, 271, 526e536 |
| $Co_2P/Mo_2C/Mo_3Co_3C$@C | 10 | 362 | 82 | 10 h at an overpotential of 360 mV | J. Mater. Chem. A 2018,6, 5789-5796 |
